# PDB-IHM: A System for Archiving and Dissemination of integrative structures

**DOI:** 10.1063/4.0000891

**Published:** 2025-10-27

**Authors:** Brinda Vallat

**Affiliations:** RCSB PDB, Rutgers University, Piscataway, NJ, USA

## Abstract

Structures of many large biomolecular assemblies are now being determined using integrative approaches, where information derived from multiple experimental and computational methods is combined to compute three-dimensional structures of macromolecular complexes. A standalone prototype data resource for integrative structures called PDB-Dev was built, based on recommendations of the Integrative and Hybrid Methods (IHM) Task Force of the Worldwide Protein Data Bank (wwPDB). This effort included developing data standards and software tools for collecting, curating, validating, visualizing, archiving, and disseminating integrative structures that span diverse spatiotemporal scales and conformational states. Building upon this foundational framework, PDB-Dev has been further expanded to handle large dynamic macromolecular systems and integrative structures that combine, for example, experimental restraints with atomic coordinates computed by machine learning algorithms. Data standards and supporting tools have also been extended to capture information about biomolecular dynamics, such as conformational transitions and related kinetic data derived from biophysical methods. Furthermore, mechanisms have been developed to validate integrative structures based on the experimental data underpinning them, including creation of methods for interoperating with external data resources such as SASBDB (sasbdb.org) and PRIDE (ebi.ac.uk/pride/) to facilitate streamlined access to experimental data for structure validation. Recently, PDB-Dev was unified with the PDB archive and rebranded as PDB-IHM (pdb-ihm.org), further promoting FAIR (Findable, Accessible, Interoperable, and Reusable) principles of data stewardship for integrative structural biology.

